# Management of Traumatic Uterine Rupture in Blunt Abdominal Trauma: A Case Report and Literature Review

**DOI:** 10.7759/cureus.8396

**Published:** 2020-06-01

**Authors:** Garrett Suchecki, Hannah Tilden, Kristina Roloff, Deepak Chandwani, Michael Neeki

**Affiliations:** 1 Emergency Medicine, Arrowhead Regional Medical Center, Colton, USA; 2 Obstetrics and Gynecology, Arrowhead Regional Medical Center, Colton, USA

**Keywords:** blunt abdominal, trauma, uterine rupture, pregnancy, cesarean delivery

## Abstract

A 27-year-old female with an 18-week pregnancy was involved in a high impact motor vehicle accident due to which she suffered a uterine rupture secondary to blunt abdominal trauma. Traumatic uterine rupture may result from blunt abdominal traumas such as those that occur during motor vehicle accidents. Prompt diagnosis is necessary to treat this complication given its quick onset and progression, and prevent potential life-threatening complications to mother and fetus. Here, we present a unique case of uterine rupture that was surgically repaired, allowing for the continuation of pregnancy.

## Introduction

Motor vehicle accidents, domestic violence, and falls are the most common causes of blunt trauma during pregnancy [1]. Uterine rupture in pregnancy due to trauma is an obstetric emergency that is potentially life-threatening to both the mother and the fetus. Rupture of the uterus can also follow misoprostol use in women with a prior uterine scar, obstructed labor, and rarely without explanation or due to blunt abdominal trauma [2-3]. Traumatic uterine rupture accounts for approximately 10% of all cases of ruptured uterus [3].

## Case presentation

**Figure 1:**
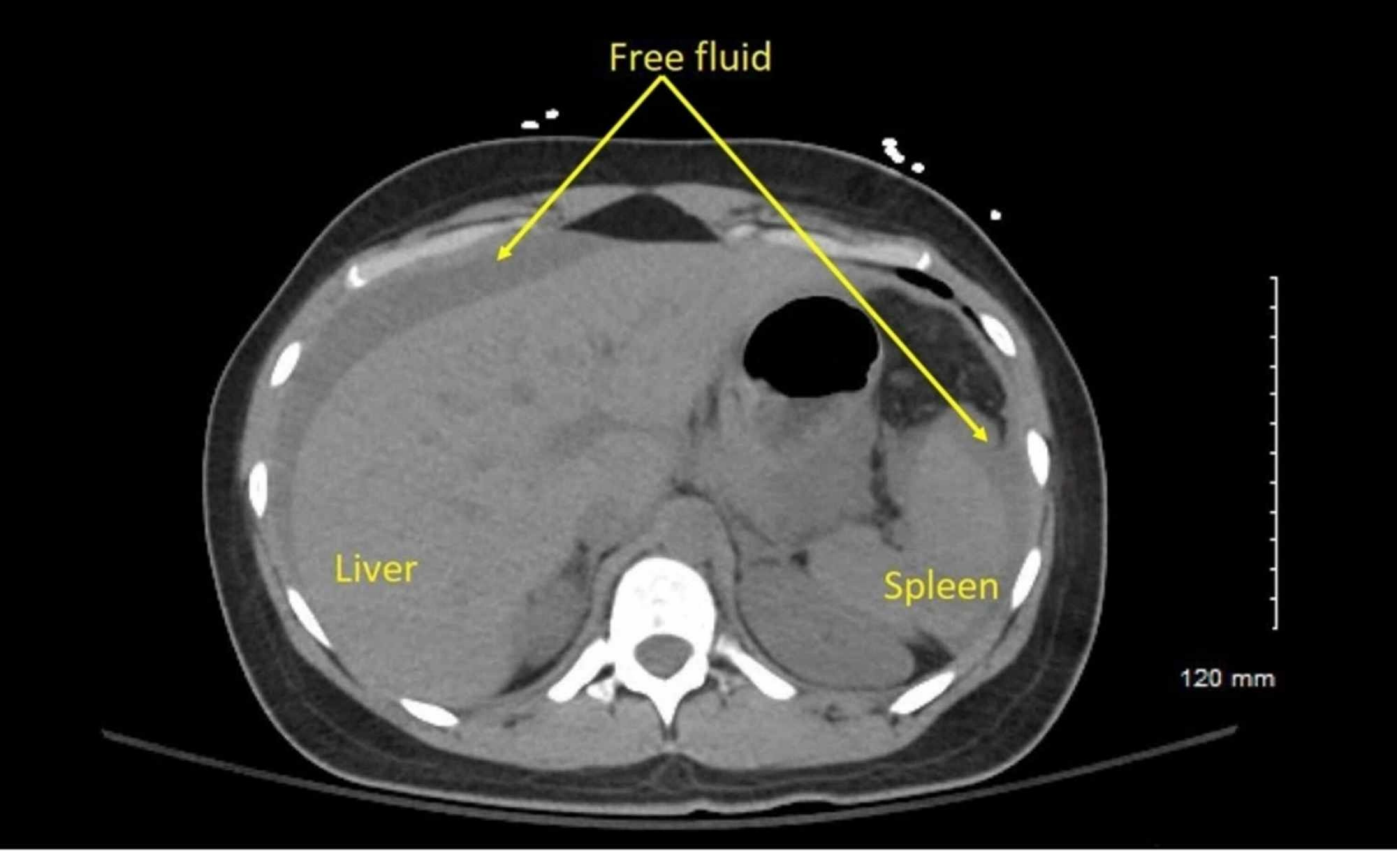
Transverse CT at level of mid-spleen with intraperitoneal free fluid.

**Figure 2:**
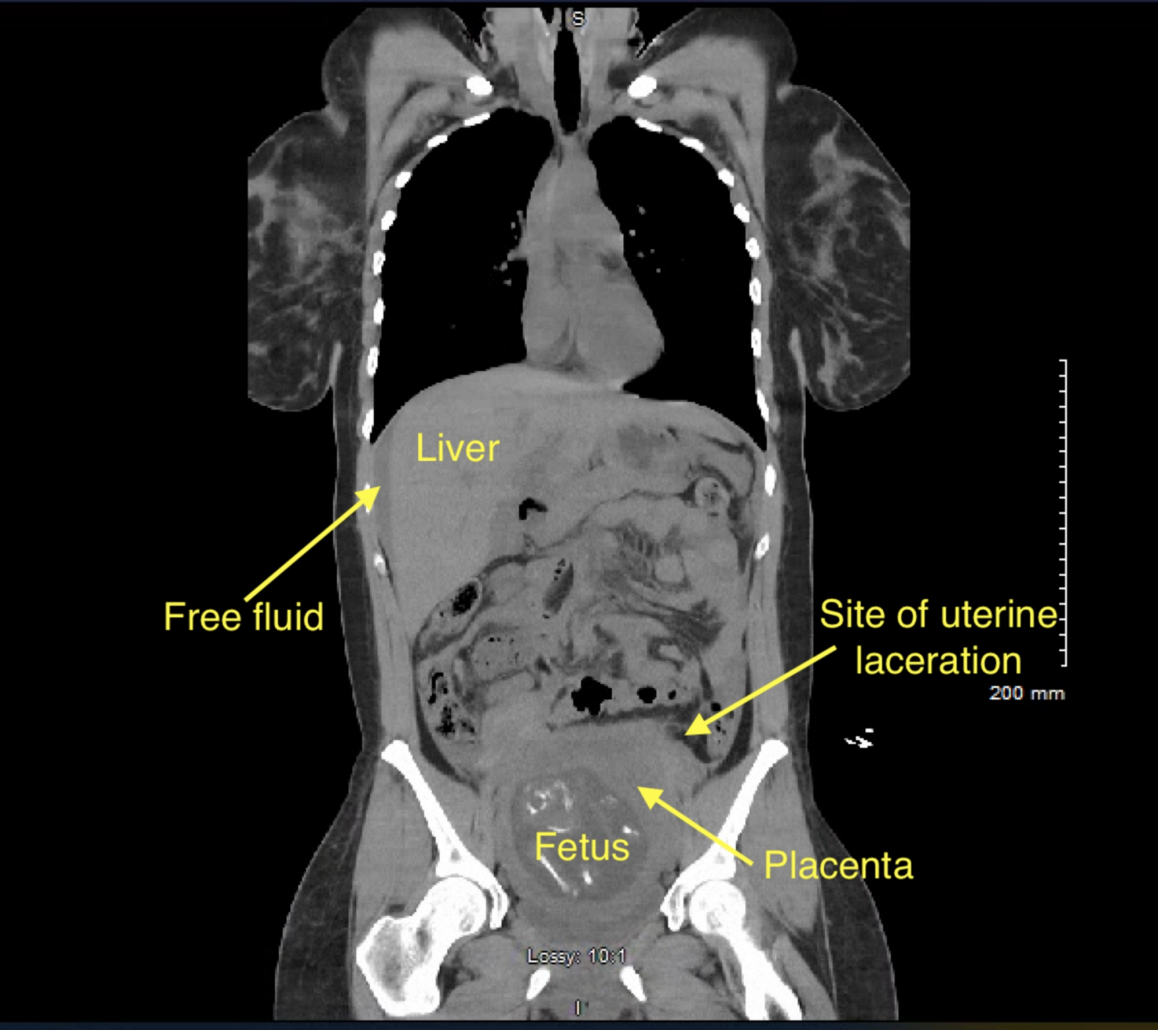
Coronal reconstruction of CT from Figure 1, also with free intra-peritoneal free fluid.

**Figure 3:**
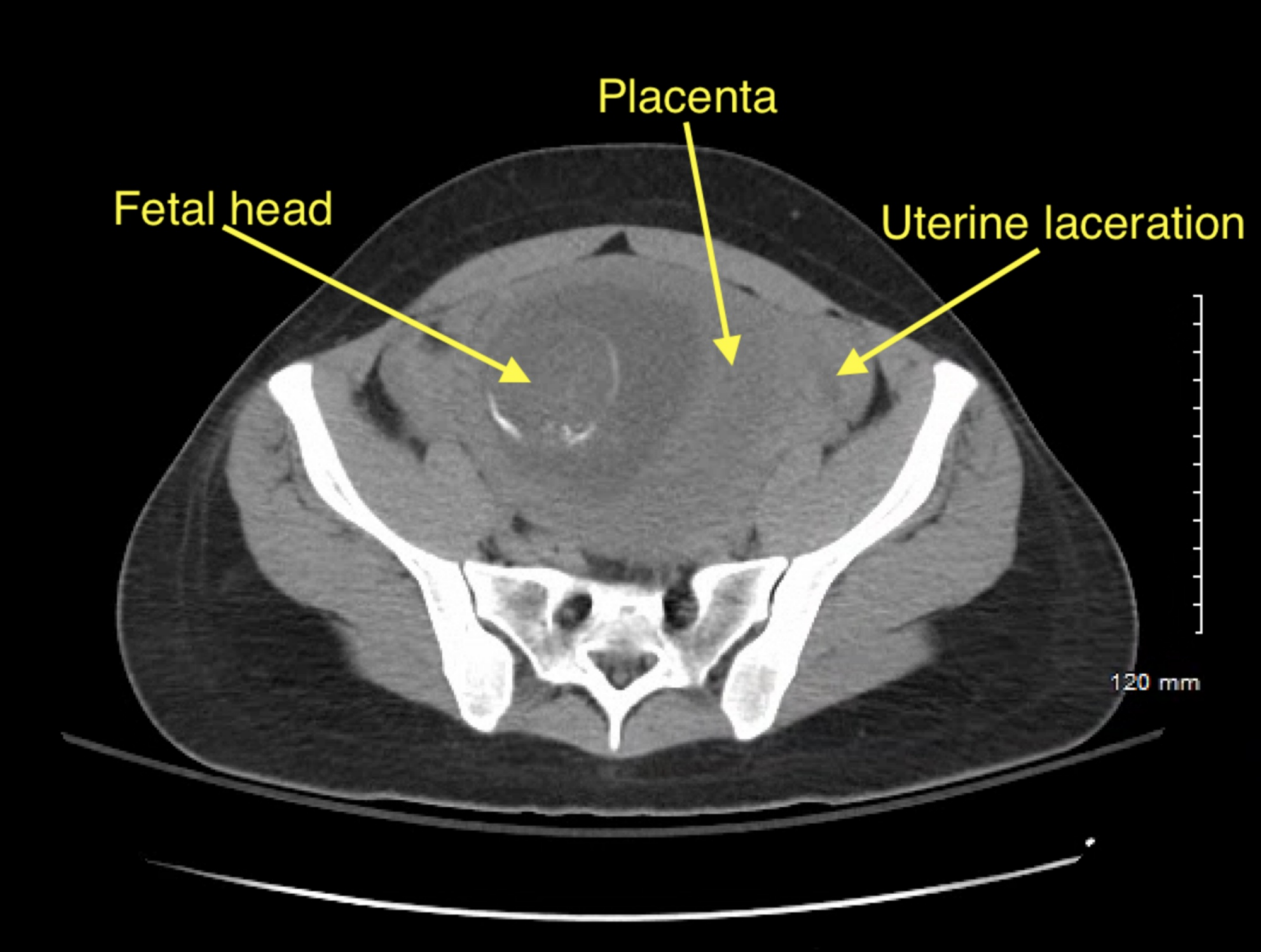
Transverse CT of pelvis with fetus within uterine cavity.

**Figure 4:**
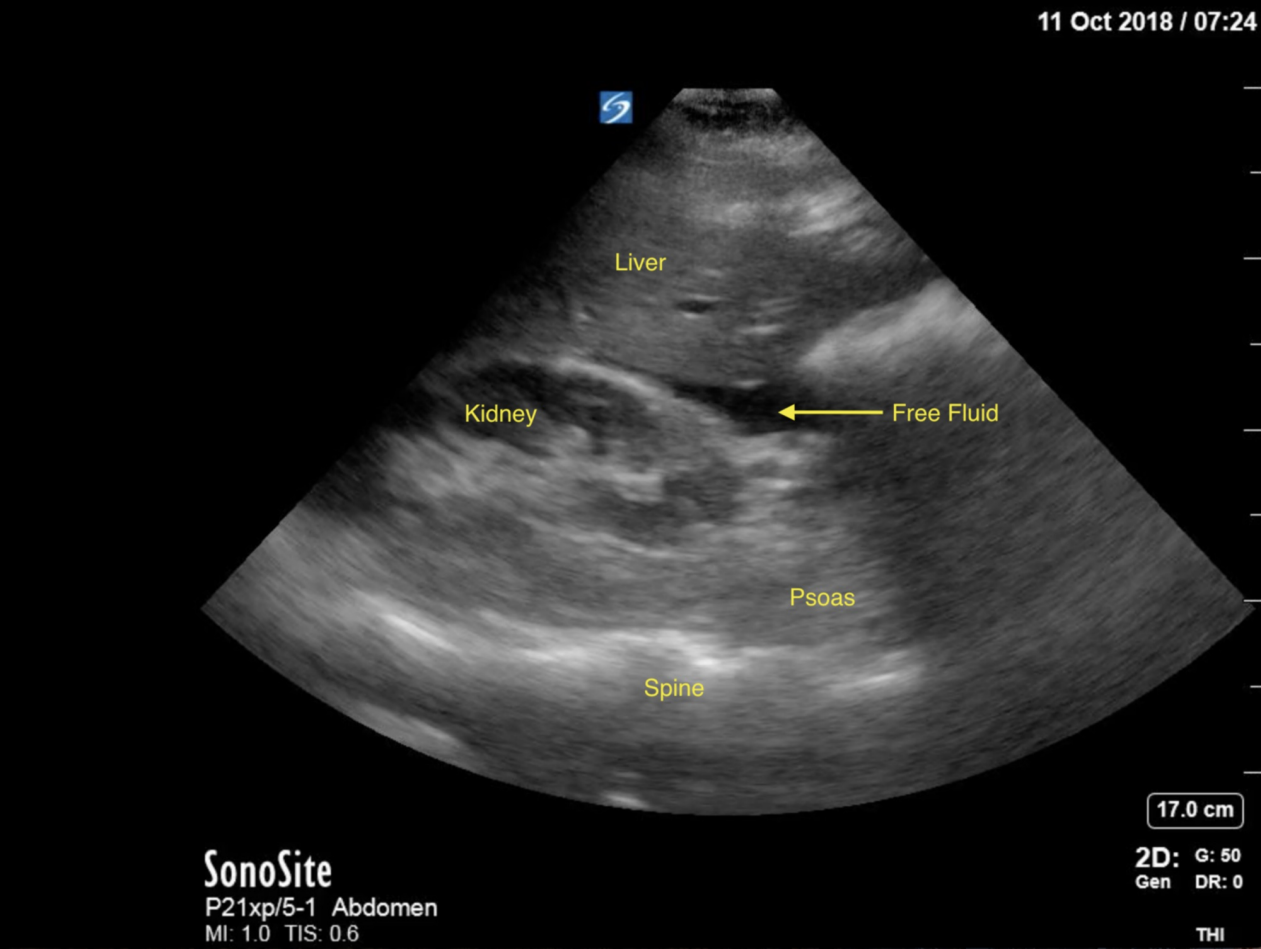
FAST scan demonstrating free fluid at the time of the motor vehicle accident.

A 27-year-old G3P1 with an intrauterine pregnancy at 18 1/7 weeks was brought to the ED following a high speed motor vehicle accident. The patient was a restrained front-seat passenger in a sedan that lost control and hit the center divider, and then was struck from the rear by a semi-truck. Airbags were deployed successfully. The patient self-extracted on site and was transported to the ED by ambulance with normal vital signs and a Glasgow Coma Scale (GCS) of 15. Her past medical history was significant for lupus, and her prior obstetric history included an uncomplicated term vaginal birth and an 11-week surgical termination. A FAST scan and limited obstetric ultrasound revealed maternal abdominal and pelvic free fluid, and an intrauterine pregnancy with fetal heart rate 130 and normal amniotic fluid with a fundal placenta (Figure 1). Non-contrast chest and abdominal CT showed moderate hemoperitoneum with no clear source of bleeding, no free air, or acute cardiothoracic injury (Figures 2-4). Uterine injury was suspected and the patient was immediately brought to the operating room for emergency exploratory laparotomy. Intraoperatively, a 5-cm right transverse fundal laceration with exposed placenta was found. There was no active bleeding from the laceration and the myometrium was re-approximated with a figure of eight sutures. Postoperatively, obstetric ultrasound showed single live intrauterine gestation consistent with 18 weeks with a breech fetal presentation. The placenta was noted to be fundal and amniotic fluid index (AFI) was found to be 15.6 cm. The patient was placed on indomethacin and oral nifedipine for prophylactic tocolysis and observed for four days without complications.

She was followed with weekly ultrasounds in outpatient clinic without complication. Her lupus remained quiescent, but mild intrauterine growth restriction without abnormal fetal Doppler studies was noted.

**Figure 5:**
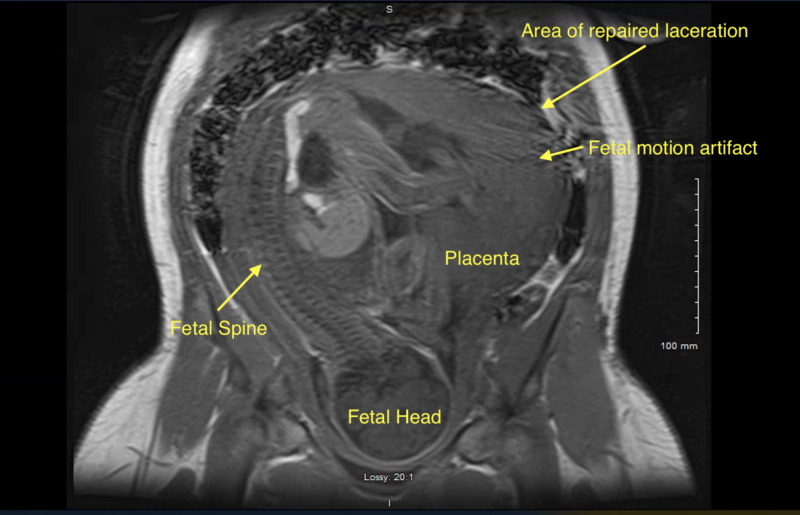
MRI at 31 4/7 weeks with motion artifact due to fetal movement, without evidence of uterine rupture.

Two months later, at 31 weeks and two days, the patient was admitted for observation due to new onset abdominal pain that she described as localized to the prior site of uterine laceration. The fetal heart rate tracing was reassuring and obstetric ultrasound showed a fetus with an estimated fetal weight of 1500 g, with asymmetric intrauterine growth restriction (IUGR), abdominal circumference <10 percentile. She was given a course of corticosteroids for fetal lung maturity. An abdominal-pelvic MRI showed no evidence of dehiscence of the uterine scar (Figure 5). Her pain improved with rest, but did not resolve. On the third day of admission the patient noted worsening and severe abdominal pain and vomiting. On physical exam, abdomen was tender to palpation primary on the left side but not distended. The fetal heart rate tracing was reassuring, though difficult to trace continuously due to patient’s movement due to pain. Given the concern for early uterine scar dehiscence, the patient underwent emergent cesarean delivery. The infant was delivered without difficulty at 31 weeks and five days. Upon inspection, previous right-sided fundus laceration was noted to be soft and indented but not ruptured. The placenta was delivered without difficulty, and the patient had no postoperative complications. She was discharged postoperative day 4.

## Discussion

Trauma is the leading nonobstetrical cause of maternal death [1]. Traumatic rupture of the uterus usually results from the most violent accidents and generally results in fetal death, severe maternal hemorrhage or other severe maternal morbidity or mortality [4].

Table 1 shows a summary of reported cases of traumatic uterine rupture during pregnancy. Six of seven cases of traumatic uterine rupture resulted in fetal demise, and the majority of reports describe motorcycle accidents as the cause of abdominal trauma [5-10]. All prior reports resulted in the delivery of the fetus at the time of surgical evaluation of the uterine laceration, and only one case resulted in live male infant via transverse C-section [11]. Total hysterectomy and laceration repair were both employed to control hemorrhage due to uterine laceration. There were no reported trends in location or size of laceration. In our case, there was a 5 cm laceration perpendicular to the direction of muscle fibers, with placental tissue extruding into the abdominal cavity at the time of repair.

**Table 1:** Summary of reported cases of traumatic uterine rupture during pregnancy. GA, gestational age; DIC, disseminated intravascular coagulation

Author	Mechanism of injury	Location of laceration	Size and direction of laceration	Fetal outcome	Maternal outcome	GA
Woldeyes et al. (2015) [8]	Motorcycle accident	Left upper uterine body	10 cm, transverse	Fetal demise	Hysterectomy, transfusion	39 weeks
Venant et al. (2013) [7]	Motorcycle accident	Fundal	5 cm	Fetal demise	Laceration repair, retroperitoneal hematoma, endmoteritis	19 weeks
Yakasai et al. (2012) [9]	Motor vehicle accident	Posterior uterine body	12 cm	Fetal demise	Hysterectomy	26 weeks
Enakpene et al. (2005) [11]	Motorcycle accident	Left lateral lower uterine segment	6 cm, longitudinal with cervix extension	Live birth, Cesarean delivery	Laceration repair	38 weeks
Belkouch et al. (2014) [5]	Motorcycle accident	Fundal	Not reported	Fetal demise	Laceration repair, DIC, hemorrhagic shock, maternal death	12 weeks
Mahajan and Kale (2008) [6]	Fall from bridge	Fundal	Not reported	Fetal demise	Hysterectomy	Term
Zydeck et al. (1995) [10]	Motor vehicle accident	Fundal	10 cm, transcornual	Fetal demise	Laceration repair, pancreatitis	37 weeks

The fetal and neonatal outcomes in the pregnancies that are delivered during motor vehicle accident admissions were poor. Approximately one-third end in perinatal death [1]. Traumatic uterine rupture necessitates immediate action toward delivery to prevent immediate demise of the fetus in most cases [12]. Fortunately, in our case, the pregnancy was able to continue past the limits of viability and a healthy preterm fetus was delivered without complications. Depending on the location of a traumatic uterine rupture, hemodynamic status of the patient, future fertility desire, availability of blood products, and experience of the surgeon, hysterectomy or repair of the laceration can be considered to achieve hemostasis [10].

Hysterotomy is performed during pregnancy in order to accomplish in utero fetal surgery -- such as in cases of fetal meningomyelocele repair [13]. In these cases, the hysterotomy is usually cut in the mid-sagittal uterus in a longitudinal fashion. Postoperatively, a tocolytic regimen of indocin and nifedipine is given to reduce stress on the uterine scar, and informed the postoperative strategy we gave in this case. Reported partial or total hysterotomy dehiscence is ~10%-11% following in utero fetal surgery [13-14]. In women with a prior classical (longitudinal) uterine incision, chances of pre-labor rupture of the scar is up to 12% [15]. Here, the traumatic uterine rupture was transverse to the muscle fibers and in the fundus. It is not known if the direction of uterine laceration (transverse or longitudinal) impacts the chance of uterine scar rupture.

## Conclusions

Rupture of the gravid uterus remains a disastrous complication of trauma. There are few case reports addressing uterine injury resulting from maternal trauma, and the majority result in fetal demise. Here, we report a unique case of continuing pregnancy with live-birth following repair of a traumatic uterine laceration with exposed placental tissue without hemorrhage. This case shows that a good outcome can be obtained with thoughtful and careful repair of a uterine laceration due to maternal trauma.
